# BrainDevo: Spatio-Temporal Gene Regulation Repository of Brain Development

**DOI:** 10.3389/fnmol.2022.799801

**Published:** 2022-03-22

**Authors:** Sathish Periyasamy, Bryan Mowry

**Affiliations:** ^1^Queensland Brain Institute, The University of Queensland, Brisbane, QLD, Australia; ^2^Queensland Centre for Mental Health Research, The University of Queensland, Brisbane, QLD, Australia

**Keywords:** brain development, gene regulation, transcriptomics, transcription factors, micro RNA (miRNAs), computational modeling, data mining and knowledge discovery, human brain

## Introduction

The field of evolutionary developmental biology has contributed to identifying the mechanisms by which the toolkit genes shape the embryo and the organism's body plan by generating spatio-temporal patterns (Gerhart and Kirschner, 2007). These genes, which control the organism's embryonic development, are ancient and highly conserved across the animal kingdom. Most of them are components of signaling networks encoding the production of transcription factors (TFs), cell adhesion proteins, cell surface receptor proteins and morphogens. Moreover, these genes function as master regulators by orchestrating other regulatory genes which regulate target/core genes involved in anatomical and physiological processes in the developing embryo. Core genes are defined as target genes having biologically interpretable roles in pathophysiology (Boyle et al., 2017; Liu et al., 2019). The deviations of core genes' regulation can contribute to variation in the developing embryo. However, these deviations are predominantly due to the mechanisms by which the toolkit genes precisely regulate the core genes' expression in space and time (Carroll, 2008). Moreover, single nucleotide polymorphisms (SNPs) in these regulatory regions can impact TF-DNA interactions and play a crucial role in regulating core gene expression.

Numerous Systems Biology approaches (e.g., clustering, classification and dynamic regulatory network analysis) and -omics data sources are available to analyse of time-series gene expression data (Bar-Joseph et al., 2012). Currently, molecular systems approaches are contributing to reconstruct the regulatory activities in stimulus-response, developmental and cell cycle studies (Bar-Joseph et al., 2012). Dynamic Regulatory Event Miner (DREM) (Schulz et al., 2012), a unique approach, integrates disparate-omics data and time-series gene expression data (Ernst et al., 2007; Schulz et al., 2012, 2013) to identify target genes regulated by TFs. Thus, contributing to the capture of time and region-specific gene regulation in developmental studies such as human brain development.

Currently there are spatio-temporal brain gene expression resources available to study the dynamics of gene expression in developing human and mouse brains. Although some human gene expression dynamics have been published, the regulatory dynamics of TFs and miRNAs have not been investigated. As TFs can have a global impact on brain development, the regulatory trajectories can provide valuable insights into gene regulation of the developing brain.

BrainDevo is an interactive database containing TFs/miRNAs and target genes involved in human brain development. The database consists of TF/miRNA regulations of genes from prenatal to postnatal stages of brain development in 16 brain regions. This interactive tool can be used to study the transcription factors regulating key stages of brain development. Genes identified from various brain development disorders can be interrogated using the interactive map of gene regulatory trajectories of the developing brain.

## Materials and Methods

### Primary Data Collection and Pre-processing

The spatio-temporal brain gene expression data was obtained from BrainSpan (Miller et al., 2014) (http://www.brainspan.org/). It consists of developmental transcriptome data (RNA-seq data; *N* > 52,000 transcripts) from post-conception to adulthood in 16 brain regions (Brainspan, 2013). Each time point for each brain region is represented by either a male or female depending on the availability of post-mortem samples. Moreover, the samples are represented by deceased individuals from diverse ancestry (Allan Brain Atlas, 2013). The transcripts include protein-coding genes, miRNA and LncRNAs. The prenatal time points include 8, 9, 12, 13, 16, 17, 19, 21, 24, 25, 26, 35, and 37 weeks and postnatal time points include 4 months, 10 months, 1, 2, 3, 4, 8, 11, 13, 15, 18, 19, 21, 23, 30, 36, 27, and 40 years. The 16 brain regions are listed in Supplementary Table 1. The regions are classified based on stages of development (i.e. Stage 1: 4–7 weeks; Stage 2A: 8–9 weeks; Stages 2B: 11 week−10 to 40 years). There were no data for stage 1. Stage 2A regions were merged with stages 2B-11 regions based on how stage 2A brain regions differentiated into various mature brain regions from stage 2B. Each time point in the analysis was represented by a single sample as, most time points in each brain region had expression data generated from a single individual.

Gene regulatory data was obtained from RegNetwork-Regulatory Network repository (http://www.regnetworkweb.org/) (Liu et al., 2015), miRWalk (Sticht et al., 2018) and miRTarBase (https://mirtarbase.cuhk.edu.cn/) (Chou et al., 2018).

### Generating Spatio-Temporal TF/MiRNA Regulatory Trajectories in Human Brain Development

We used iDREM for integrated analysis of time-series gene expression data and static regulatory interaction data to reconstruct dynamic developmental activities regulated by TFs and miRNAs (Ding et al., 2018). This incorporates developmental transcriptome data obtained from post-conception to adulthood (i.e., prenatal and postnatal) in 16 brain regions.

The temporal gene expression files for each of the 16 brain regions were extracted from the BrainSpan dataset. TF-gene interaction file was derived from the RegNetwork database. The default gene annotation sources provided by iDREM were used. A human gene cross-reference file was used to map synonymous gene names. The RNA-seq expression data were log normalized before the analysis using iDREM. The miRNA brain gene expression data were also log normalized before the analysis. The miRNA-gene regulatory data provided by iDREM was used to predict the miRNAs involved in spatio-temporal brain development. The miRNAs with no expression data were filtered out. The default filter options were used and for search options, the recommended parameters for Convergence Likelihood %: 0.01 and Minimum Standard Deviation: 0.2. The default parameters were used for gene ontology annotations and analysis. The analysis was conducted in a high-performance computing cluster and it took 2–3 weeks per brain region. TFs with a *p* < 0.001 were listed as significant regulators. The users can use the significant regulators (*p* < 0.001) in every brain region to map to the disease-associated TFs/miRNAs obtained from the genome-wide significant GWAS loci of psychiatric and other brain disorders.

## Braindevo Data Availability and Usage

### BrainDevo Data Deployment

**Figure 1:**
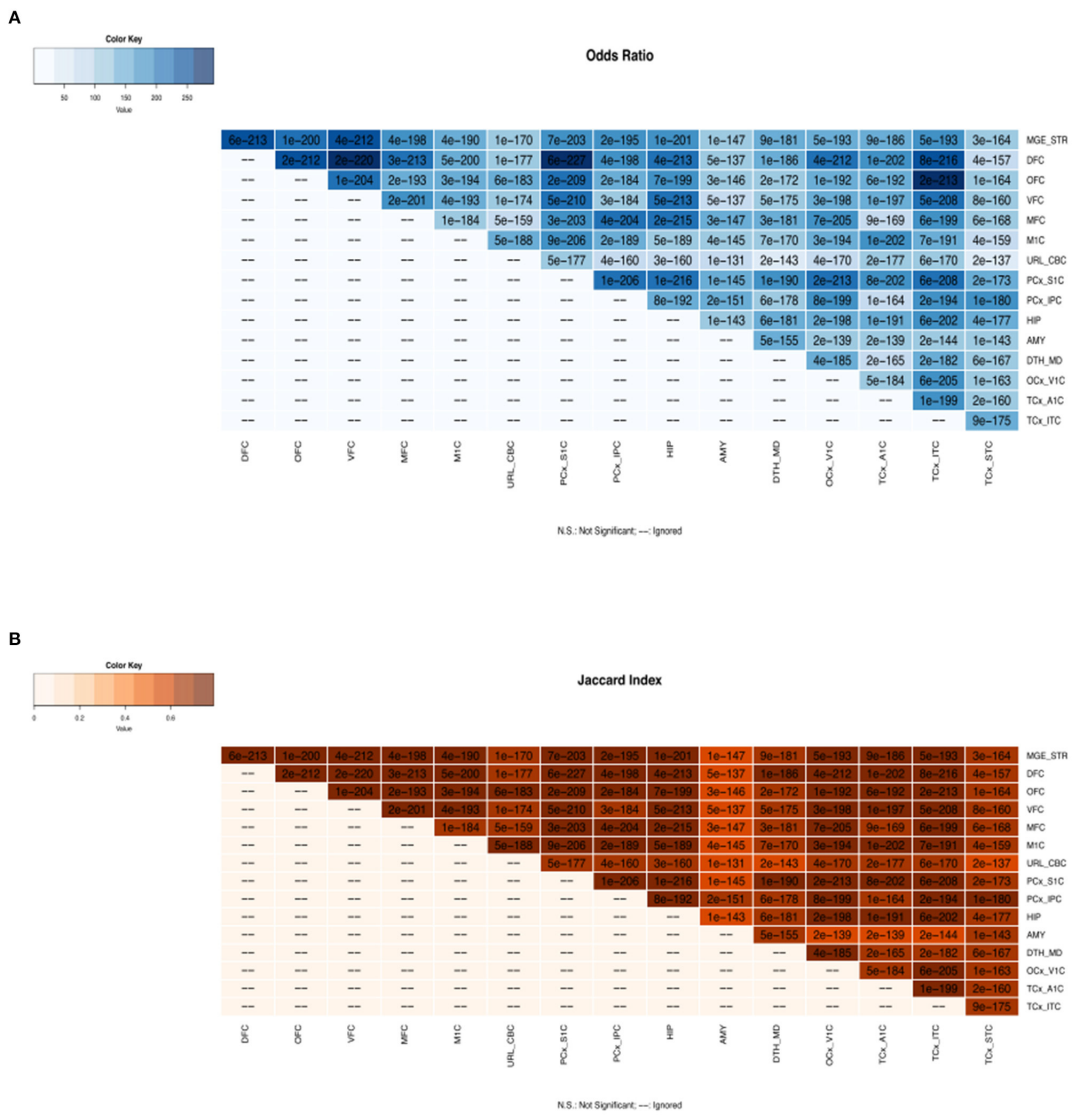
Regulatory gene overlap analysis across all brain regions. **(A)** The color key represents the odds ratios and the significant *p*-values are superimposed on the grids. The odds ratio and *p*-values indicate that there is strong association of gene regulators across brain regions. **(B)** The color key represents the Jaccard index and the significant *p*-values are superimposed on the grids. The Jaccard index measures the similarity of gene regulators across brain regions. MGE_STR, Medial ganglionic eminence_Striatum; DFC, dorsolateral prefrontal cortex; OFC, orbital frontal cortex; VFC, ventrolateral prefrontal cortex; MFC, medial prefrontal cortex; M1C, primary motor cortex; URL_CBC, upper rhombic lip_Cerebellar cortex; PCx_S1C, parietal neocortex_Primary somatosensory cortex; PCx_IPC, parietal neocortex_Inferior parietal cortex; HIP, hippocampus; AMY, amygdaloid complex; DTH_MD, dorsal thalamus_Mediodorsal nucleus of thalamus; OCx_V1C, occipital neocortex_Primary visual cortex; TCx_A1C, temporal neocortex_Primary auditory cortex; TCx_ITC, temporal neocortex_Inferolateral temporal cortex; TCx_STC, temporal neocortex_Superior temporal cortex.

We have developed and deployed a data source to identify transcriptional and post-transcriptional regulators in 16 brain regions during human brain development. There was a significant similarity of TFs involved in human brain development across all brain regions (see Figure 1). Moreover, we observed substantial gene regulatory activities during the prenatal stages of brain development. While TFs are shown to be involved in bifurcation points miRNAs are shown to be regulating paths between bifurcation points. We provide a resource comprising TFs and miRNAs regulating 16 brain regions at various brain developmental stages. These results could lead to studies of gene regulation in healthy brain development and other neurodevelopmental disorders (Supplementary Table 1).

### The User Interface

The interactive visualization interface for each brain region was generated using iDREM during the analysis. The interface (see Supplementary Figure 1) for the predicted model for each of the 16 brain regions are available at https://doi.org/10.48610/5f24ed4 (Periyasamy and Mowry, 2021). The installation will require uncompressing the downloaded file and opening idrem_results.html under each directory representing a brain region. The installation of iDREM is not required for visualizing the interface, as idrem_results.html will dynamically generate an interactive visualization interface to interrogate transcription factors and genes. For detailed functionalities, please refer iDREM user manual. The users could identify key TFs and miRNAs responsible for each bifurcation point from the interface. By clicking a node, the users could identify the downregulated, upregulated and non-expressed regulators (see Supplementary Figure 2). By clicking a node (after selecting the “genes assigned to the path” option under the global config panel), the users could observe the gene list assigned to a path (see Supplementary Figure 3). The users can use the regulatory panel to investigate a TF of interest in human brain development (see Supplementary Figure 4) and the expression panel to investigate the gene expression path and pattern during brain development.

### Applications of BrainDevo

TF and miRNAs identified in various genetic studies such as rare or common variant association studies, can be interrogated using BrainDevo. The users will be able to identify the timepoint at which a TF of interest impacts brain development. This repository will also be useful for brain developmental biologist, interested in identifying TF/miRNAs and their target genes at a particular time point and brain region.

## Conclusion

A data resource to identify transcriptional and post-transcriptional regulators in 16 brain regions during human brain development has been deployed for investigating psychiatric and brain developmental disorders.

## Data Availability Statement

Publicly available datasets were analyzed in this study. BrainDevo is freely available at https://doi.org/10.48610/5f24ed4. The users will be able to download the compressed dataset in the links section of the UQ eSpace.

## Ethics Statement

Ethical review and approval were not required for the study on human participants in accordance with the local legislation and institutional requirements.

## Author Contributions

SP was involved in data collection, pre-processing, and analysis. SP and BM were involved in reviewing the results and writing the manuscript. Both authors contributed to the article and approved the submitted version.

## Funding

This study was supported by Grant No. 1047956 from the Australian National Health and Medical Research Council (NHMRC) (BM).

## Conflict of Interest

The authors declare that the research was conducted in the absence of any commercial or financial relationships that could be construed as a potential conflict of interest.

## Publisher's Note

All claims expressed in this article are solely those of the authors and do not necessarily represent those of their affiliated organizations, or those of the publisher, the editors and the reviewers. Any product that may be evaluated in this article, or claim that may be made by its manufacturer, is not guaranteed or endorsed by the publisher.
