# Supplementary material for: BrainDevo: Spatio-Temporal Gene Regulation Repository of Brain Development
Source: Front Mol Neurosci. 2022 Mar 22;15:799801. doi: 10.3389/fnmol.2022.799801 (PMC8981586; doi:10.3389/fnmol.2022.799801)
Supplement: Supplementary file 2 [file Data_Sheet_1.DOCX]

Supplementary Material

# Supplementary Data

BrainDevo is freely available at [https://doi.org/10.48610/5f24ed4]. The users will be able to download the compressed dataset in the links section of the UQ eSpace. The uncompressed file will contain sub-directories representing each brain region.

# Supplementary Figures and Tables

## Supplementary Figures


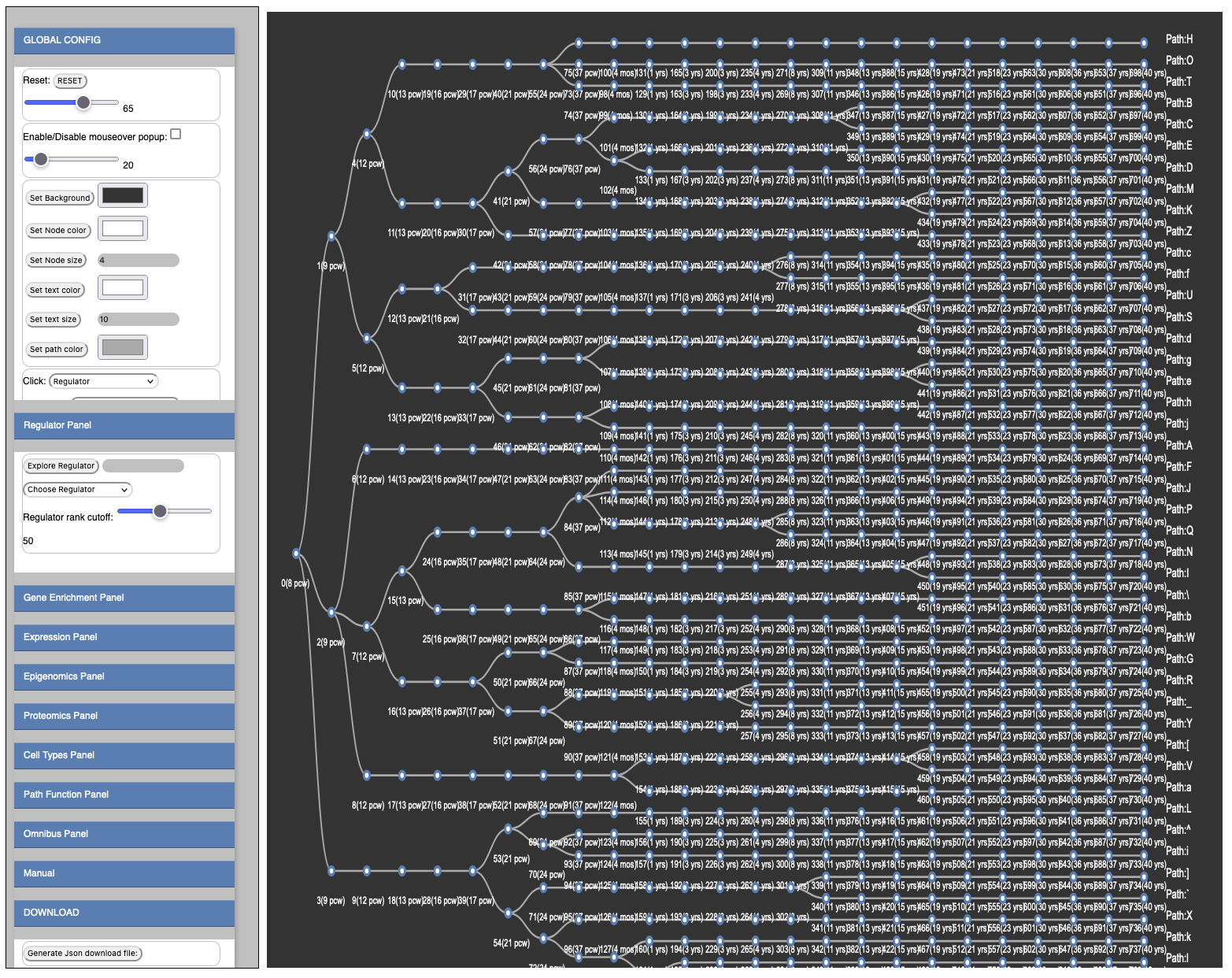


Supplementary Figure. 1: iDREM interactive visualization. The visualization of a single brain developmental region across various timepoints


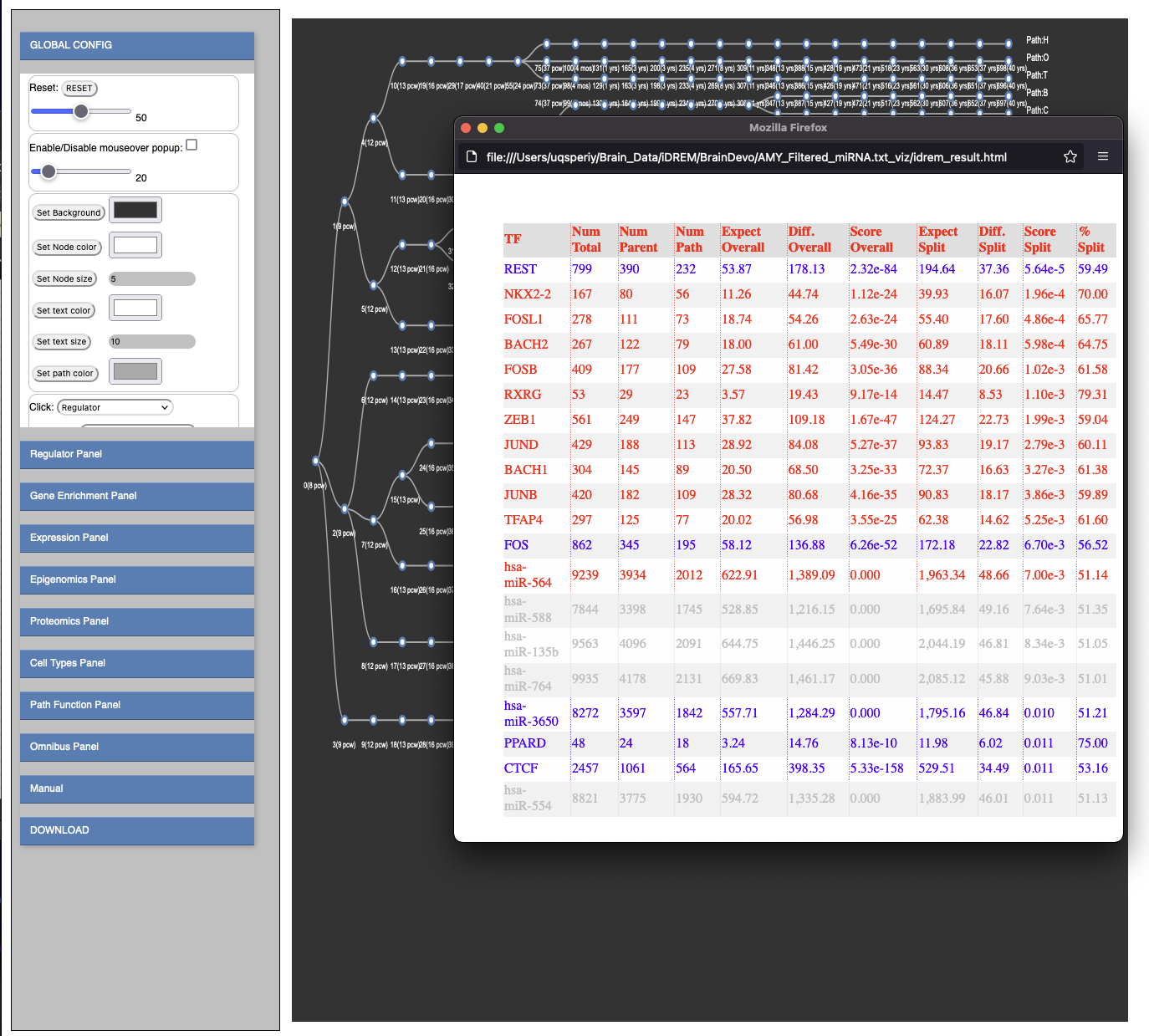


Supplementary Figure. 2: By clicking a node, the users could identify the downregulated, upregulated and non-expressed regulators. The regulator option will have to be selected as the click function under global config panel


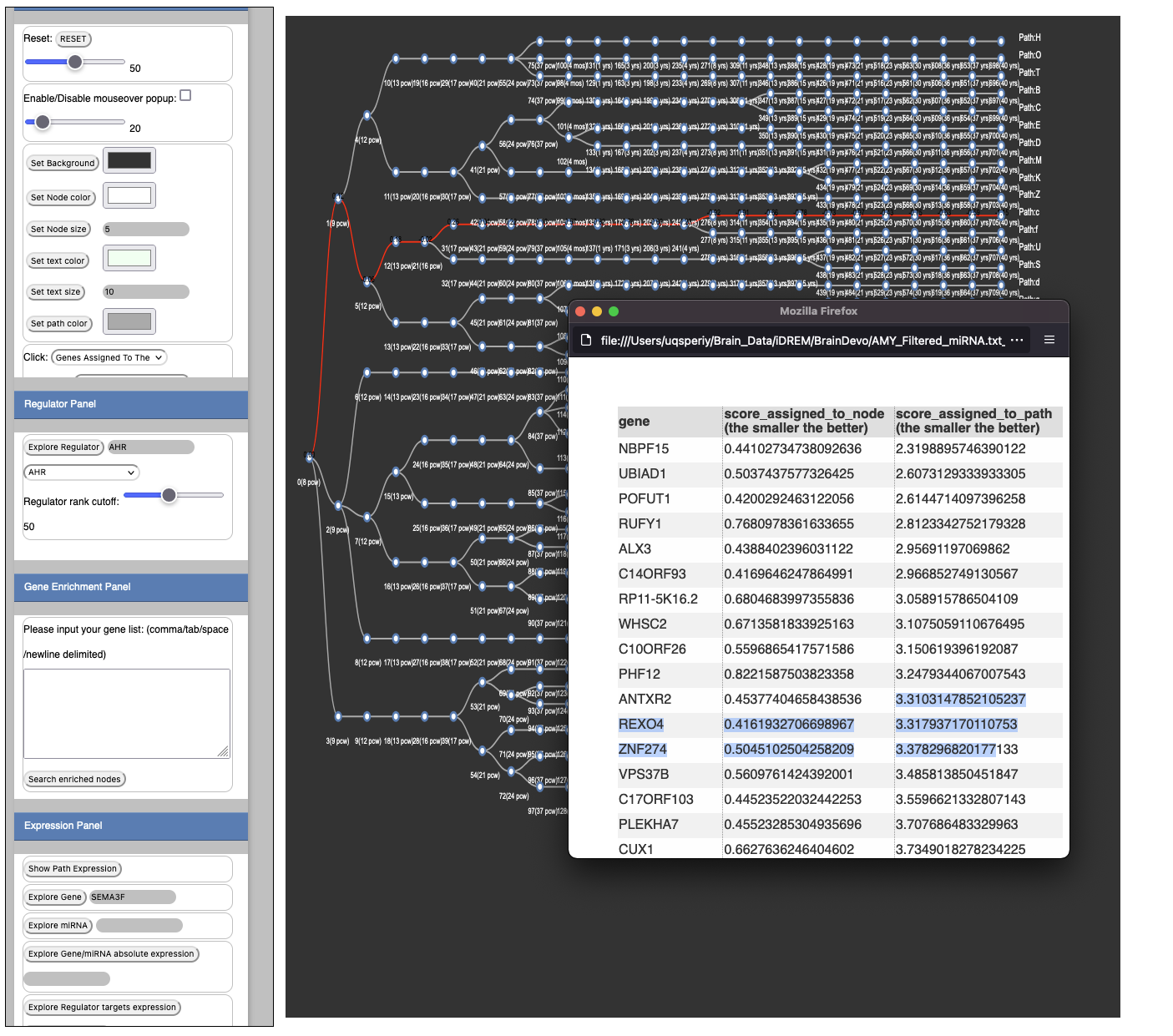


Supplementary Figure. 3: By clicking a node, the users could identify the genes involved in path associated to the node. The “genes assigned to the path” option will have to be selected as the click function under the global config panel


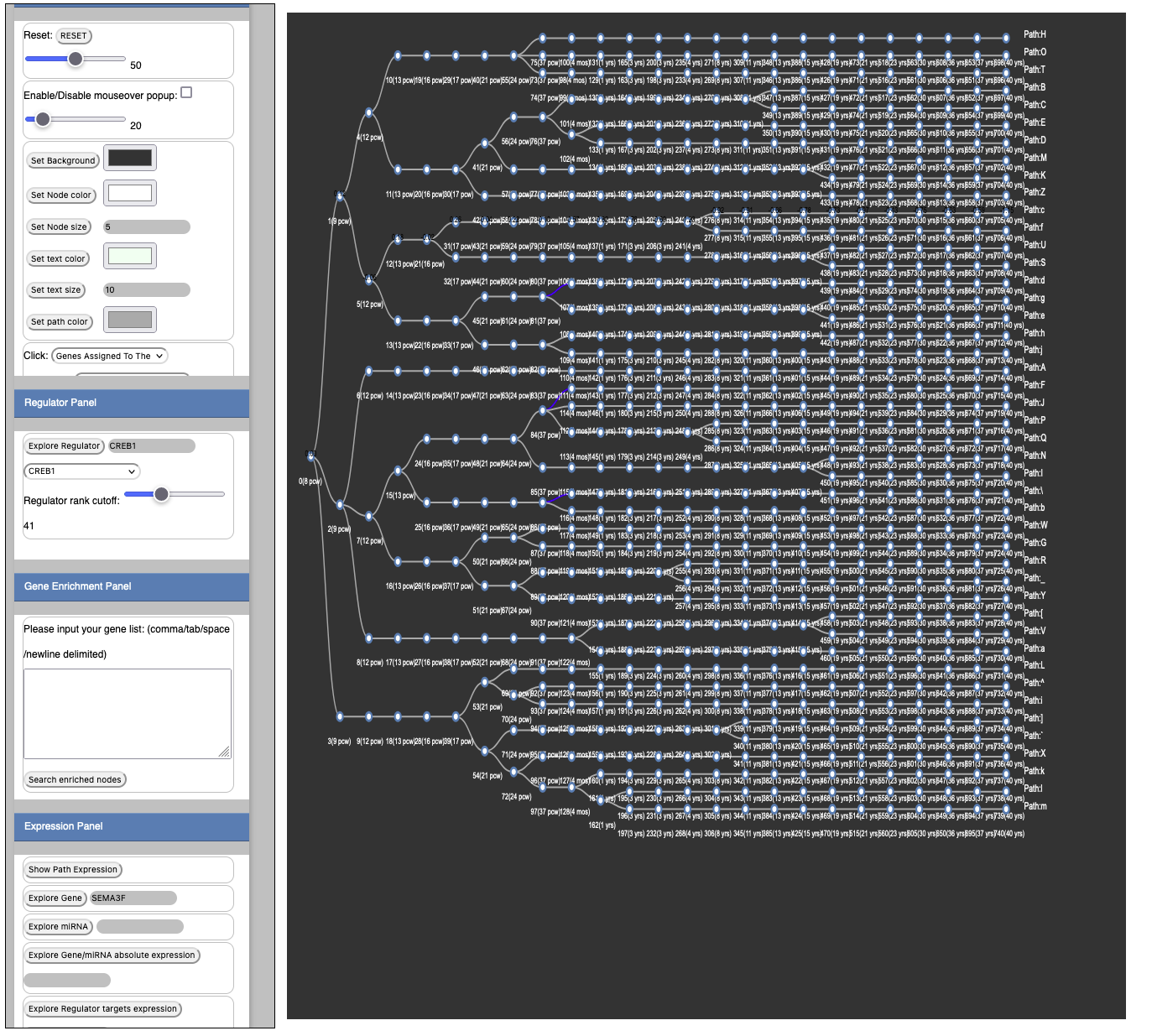


Supplementary Figure. 4: Exploring regulators. The transcription factor *CREB* is shown (in blue) to be active in three paths between 37 weeks to four months of prenatal brain development

## Supplementary Tables

The details of the regulators involved in 16 brain regions are listed in Supplementary Table 1: Supplementary_Table1_Spatio_Temporal_Brain_Gene_Regulators.xlsx
